# Editorial: Towards an Understanding of Tinnitus Heterogeneity

**DOI:** 10.3389/fnagi.2019.00053

**Published:** 2019-03-19

**Authors:** Christopher R. Cederroth, Silvano Gallus, Deborah A. Hall, Tobias Kleinjung, Berthold Langguth, Antonello Maruotti, Martin Meyer, Arnaud Norena, Thomas Probst, Rüdiger Pryss, Grant Searchfield, Giriraj Shekhawat, Myra Spiliopoulou, Sven Vanneste, Winfried Schlee

**Affiliations:** ^1^Department of Physiology and Pharmacology, Karolinska Institutet, Biomedicum, Stockholm, Sweden; ^2^Department of Environmental Health Sciences, Istituto di Ricerche Farmacologiche Mario Negri IRCCS, Milan, Italy; ^3^Nottingham Biomedical Research Centre, National Institute for Health Research, Nottingham, United Kingdom; ^4^Hearing Sciences, Division of Clinical Neuroscience, School of Medicine, University of Nottingham, Nottingham, United Kingdom; ^5^University of Nottingham Malaysia, Semenyih, Malaysia; ^6^Department of Otorhinolaryngology, University Hospital Zurich, University of Zurich, Zurich, Switzerland; ^7^Department of Psychiatry and Psychotherapy, University of Regensburg, Regensburg, Germany; ^8^Dipartimento di Giurisprudenza, Economia, Politica e Lingue Moderne, Libera Università Maria SS. Assunta, Rome, Italy; ^9^Neuroplasticity and Learning in the Healthy Aging Brain (HAB LAB), Department of Psychology, University of Zurich, Zurich, Switzerland; ^10^Laboratoire Neurosciences Intégratives et Adaptatives, Aix-Marseille Université, Marseille, France; ^11^Department for Psychotherapy and Biopsychosocial Health, Danube University Krems, Krems an der Donau, Austria; ^12^Institute of Databases and Information Systems, Ulm University, Ulm, Germany; ^13^Section of Audiology, Eisdell Moore Centre, The University of Auckland, Auckland, New Zealand; ^14^Center for Learning and Teaching (CfLAT), Auckland University of Technology, Auckland, New Zealand; ^15^Faculty of Computer Science, Otto-von-Guericke-University Magdeburg, Magdeburg, Germany; ^16^Lab for Clinical and Integrative Neuroscience, School of Behavioral and Brain Sciences, The University of Texas at Dallas, Richardson, TX, United States; ^17^Institute for Global Brain Health and Institute for Neuroscience, Trinity College Dublin, Dublin, Ireland

**Keywords:** tinnitus, heterogeneity, neuroscience, genetic, animal model, hearing

Despite being a common condition that affects nearly 15% of the population, and despite much research progress made in the recent years, tinnitus remains a scientific and clinical enigma. Subjective tinnitus is defined as a phantom perception of a tone or noise in the absence of any physical source. It is known to be a heterogeneous condition, both in the way of manifestation and of generation. In general, “heterogeneity” describes the fact that there is a non-uniform appearance of a substance, organism, or disease. Whenever there is a non-uniformity in at least one quality, we can call it “heterogeneous.” Tinnitus patients differ on at least four dimensions: First, tinnitus patients may present diverse clinical profiles with respect to the ***perception***of tinnitus (e.g., laterality of tinnitus, tinnitus pitch, ringing, buzzing, hissing, or cricket sounds). Additionally, tinnitus can be occasional or permanent, acute or chronic, pulsatile, or constant. Second, while there are multiple ways of perceiving tinnitus, it is also associated with multiple ***causal risk factors***—hearing loss, temporomandibular joint disorder, and aging being among the most common ones. There are also numerous ***related comorbidities***that add to the complex clinical picture of tinnitus (e.g., hyperacusis, depression, sleep disorders, headache, concentration problems). A third dimension is the associated tinnitus ***distress***, the psychological reaction to the ongoing tinnitus perception; it can differ largely among patients. Fourth, there is a large variation of ***treatment responses***of the tinnitus patients.

With the heterogeneity in these four dimensions, we describe a diversity of observable qualities that can be investigated with the currently available research. The current interpretation of this observed heterogeneity is that several different tinnitus subtypes may exist and that these different subtypes may have different etiologies, different clinical profiles and different treatment responses. So far, the number of the potentially existing tinnitus subtypes is not known, nor the diagnostic criteria to identify them. The situation gets even more complex when we consider patients with a combination of several subtypes.

This heterogeneity culminates in the challenge for tinnitus treatment: A uniformly effective treatment for all tinnitus patients is unlikely. For each individual patient, a personalized treatment plan has to be developed, considering the tinnitus profile, the comorbidities, the psychological distress and the previous treatment experiences of the patient. To solve this clinical enigma, conceptual models of tinnitus are needed to develop innovative solutions for personalized medicine.

**The aim of this research topic** is to investigate the challenge of tinnitus heterogeneity by involving multiple disciplines ranging from neuroscience, neurology, genetics, audiology, otolaryngology, psychology, psychiatry, pharmacology, epidemiology, medical informatics, data mining, and statistics. The main idea is to move away from an abstract view of tinnitus toward an detailed understanding of what could constitute tinnitus subtypes in view of improving fundamental knowledge and ultimately lead to optimized therapeutic interventions. Within this topic, current knowledge is reviewed and new theories of tinnitus generation are proposed, animal models are used to understand the neural correlates better, audiological, and psychological aspects are explored, neuroimaging techniques investigate the involved brain networks, new questionnaire instruments are developed and others adapted to new languages, genome-wide associations are pioneered, mobile applications are used to explore tinnitus on new time-scales, and, finally, multiple therapeutic approaches are tested.

**Statistics on this research topic:** The research topic was open between February 18, 2016 and October 29, 2017. It received 96 submissions by 335 authors. 79 submissions were finally accepted after a rigorous and constructive reviewing process. During the phase in which the topic was active, it received over two million views.

**Overview of this research topic:** To cover these themes, this Frontiers research topic starts with several retrospective reviews of the current status of the field, before introducing a number of emerging hypotheses on how tinnitus is generated, and how it is experienced by individuals when it is persistent. The first chapter on the research performed on animals addresses the validity of existing models and improves the sensitivity of outcome measures. The second chapter addresses audiological and psychological aspects to tinnitus, as well as the accompanying co-morbidities. The third chapter concerns the instruments and technologies to assess tinnitus, ranging from questionnaires, neuroimaging methods, and mobile applications. The fourth chapter reviews existing clinical guidelines and describes the recent advances in tinnitus therapy including cognitive behavioral therapy (CBT), sound therapy, cochlear implants, electric stimulation, and repeated transcranial magnetic stimulation. Finally, the fifth chapter refers to advances on tinnitus therapies. These are briefly described below:

## Chapter 1: Hypotheses and Theories

There are many subtypes of tinnitus, and in at least one subtype, the tinnitus can be considered as a symptom of the aging ears and brain. Some theories of tinnitus take a gerontological perspective. Age-related hearing loss and tinnitus can go hand in hand and one of the most well-established theories considers tinnitus as the perceptual consequence of neuronal hyperactivity in the central auditory system; emerging after loss of normal input from the ear. Computational modeling can be one productive method for exploring the nature of the underlying neural signaling pathway based on certain assumptions about spatial and temporal dynamics of excitatory and inhibitory synaptic weighting and corresponding action potential activity. In their modeling approach, Krauss et al. examine stochastic resonance. This is an adaptive mechanism whereby weak sub-threshold signals can still be detected and transmitted upwards if internal noise is added. The authors present a thought-provoking hypothesis that neuronal hyperactivity is a “side effect” of stochastic resonance in a system whose main purpose is to optimize transmission of impoverished sound information.

Animal models provide a second productive approach to investigating the underlying neural signaling pathway in the aging brain. Ruan et al. treat new ground by considering the role of the cholinergic system. Their hypothesis that cholinergic innervation of various brain structures provides a link between tinnitus seen in age-related hearing loss and age-related cognitive impairment is supported by animal literature, as discussed in the paper.

One of the challenges in this field has been to provide a neuroscientific account of tinnitus that adequately explains the common perceptual experience (i.e., the conscious perception of a sound that does not have an external source), and yet at the same time has the flexibility to account for the heterogeneity in etiology, comorbidity, psychosocial impact, and such like. Ghodratitoostani et al. rose to this challenge. The neurofunctional tinnitus model described in this article has many elements in common with other contemporary models, and is applied by the authors to interpret clinical phenomena.

Of course, it is preferable to have a treatment whose therapeutic efficacy can be understood within the context of a theoretical model, but this is not a prerequisite for treatments to be clinically beneficial. Neurofeedback is an intriguing approach to modulate the tinnitus-related brain activity patterns. A review by Güntensperger et al. concisely summarizes the progress made in hypothesis testing, experimental design and neurofeedback algorithms. A clear presentation of the limitations sets out a challenge to provide insights on the variability in efficacy that is observed across individuals.

Despite the heterogeneity of tinnitus, somatic or somatosensory tinnitus is generally presented as if it were a distinct subtype associated with cross-modal brain connectivity. Haider et al. provided a great service to the community by systematically mapping out the literature on somatosensory tinnitus. This useful guide identified what is currently known with respect to its pathophysiology, diagnosis and treatment. What is particularly striking from the descriptions is in fact the bewildering variety of clinical presentations and potential treatment options of somatosensory tinnitus. It is therefore both relevant and timely that an international consensus among expert scientists and clinicians has been recently sought on what are the important diagnostic criteria for somatosensory tinnitus (Michiels et al., 2018). From this work, somatosensory tinnitus is not seen as a specific category of tinnitus, but more as a factor that can influence a patient's tinnitus to a greater or lesser degree. Based on more and more detailed knowledge about the involvement of the somatosensory system in tinnitus pathophysiology, a systematic review summarizes the influence of physical therapy on tinnitus (Michiels et al.).

A research topic provides the perfect channel for gathering different perspectives and for representing the true multi-disciplinarity of tinnitus research. In this regard, the original research article by Dauman et al. was particularly refreshing because it took a holistic psychosocial perspective on tinnitus, in stark contrast to the somewhat more conventional and reductionist methodology that characterized a majority of the papers in the collection. In this paper, the authors boldly delve deep into the personal experience of tinnitus to explore how each participant constructs his/her own meaning about what it is to live with a bothersome tinnitus. No previous study based on open-text responses has provided such a rich narrative about the issue of heterogeneity of the lived experience. For example, a recent review found that out of 86 studies (16,381 patients) which assessed the patient experience, only eight studies asked open questions (885 patients) (Hall et al., 2018). For the Editors, it brought the all-important emotional dimension of tinnitus under scientific scrutiny.

## Chapter 2: Insights on Neurobiological Mechanisms and Genetics: From Animals to Humans

Animal studies using micro-electrode recordings have shown that noise trauma (known to produce tinnitus in human subjects) is followed by neural hyperactivity at several stages of the auditory system, from the cochlear nucleus to the auditory cortex (Eggermont). Hesse et al. found in the inferior colliculus that the increased spontaneous firing caused by noise exposure was greater in CBA/Ca mice exposed to 100 dB (showing minimal hearing loss from ABRs or “hidden” hearing loss) compared to the 105 dB exposed group (showing small hearing loss), supporting the idea of a non-linear response between low and high threshold cochlear fibers to noise damage. When performing unilateral cochlear nerve section, Tighilet et al. found that this form of sensory deafferentation caused a dramatic reduction of the KCC2 co-transporter density in the cochlear nucleus suggesting that GABA may be less inhibitory (or even excitatory) after a cochlear lesion (Tighilet et al.) and suggesting a potential new target for pharmacological treatment of tinnitus. Using gas chromatography and mass spectrometry (GC/MS), He et al. describe the impact of noise trauma on the metabolite profiling in different brain areas, which could serve in characterizing animals with tinnitus.

The gap pre-pulse inhibition of the acoustic startle (GPIAS) is the method of choice for many animal studies on tinnitus. This approach does not require conditioning of the animals prior to the experiment and therefore saves time and resources. With the aim to further optimize this method, Schilling et al. developed a new statistical approach for the analysis of the GPIAS data in Mongolian gerbils, which is based on the fact that the amplitude ratios are approximately lognormally distributed. This allows a new statistical test that is independent from the number of repetitions of the measurement. Using GPIAS to reveal tinnitus, Longenecker et al. found that only 30% of CBA/CaJ mice exposed unilaterally to noise may present tinnitus although all of them showed an increase in spontaneous firing rate, and no change in bursting activity (Longenecker and Galazyuk). The authors suggest that neither neural hyperactivity nor bursting activity are strict neural correlates of tinnitus. However, the degree of inhibition during GPIAS in CBA mice is small and offers little dynamic range to infer the presence of tinnitus. Yu et al. found that the inhibition is more effective in C57BL/6J mice and can be improved even more when the delay between the gap and the startle stimulus is short. Using these improved parameters in combination with c-Fos activity mapping after the exposure to different sound stimuli, the neural circuits controlling GPIAS were found to differ from those regulating pre-pulse inhibition (Moreno-Paublete et al.).

Such refinements in GPIAS helped revealing a role of the glutamate aspartate transporter GLAST in salicylate-induced tinnitus (Yu et al.) This points at a first gene potentially involved in tinnitus using animal models. In this respect, greater knowledge of the genetic influences on tinnitus generation and persistence in humans may not only help developing a better understanding of the mechanisms and optimize the classification of patients, but could also contribute in the development of drugs to silence tinnitus (Lopez-Escamez et al.). In this respect, while the knowledge on the genetic basis of tinnitus is poorly understood (Vona et al.), a pilot genome-wide assocation study (GWAS) on 167 tinnitus subjects and 749 controls showed an enrichment in oxidative stress, endoplasmatic reticulum (ER) stress, and serotonin reception mediated signaling although it did not identify any significant association (Gilles et al.). A genotyping study on 78 individuals suggests that *GRM7* rs11928865 could be used as a biomarker for tinnitus severity (Haider et al.). The increasing evidence of genetic influences on tinnitus emphasizes the need of creating large biobanks in ENT clinics and merge efforts to increase statistical power (Cederroth et al.).

## Chapter 3: Auditory and Psychological Characteristics Contributing to Tinnitus Heterogeneity

Audiological factors contribute to tinnitus heterogeneity. Milloy et al. undertook a scoping review of the Auditory Brainstem Response (ABR) in tinnitus participants and found a large variation in measured latencies and amplitude of the earliest component of the ABR (wave I), likely due to a broad variation in methods, study population, sample size, and potentially the definitions of tinnitus. In a case report, Londero et al. monitored symptoms after a tinnitus-inducing acoustic shock and found abnormal tympanic membrane appearance suggestive of abnormal middle ear muscle activity causing local chronic inflammation. The authors suggest that the combination of local inflammation and neural response originating from this type of injury could result in otalgia and tinnitus, in absence of hearing loss. Ueberfuhr et al. found that low frequency sound exposure and Ménière's disease was not exclusively followed by low frequency or noise-like tinnitus, indicating a variation in the underlying pathology.

The availability of large databases generated new insights into the role of hearing in tinnitus generation. Indeed, using data from 37,661 patients, Gollnast et al. found that auditory thresholds were lower for young tinnitus patients than matched non-tinnitus patients. Thanks to data from the Tinnitus Research Initiative (*n* = 2,838 patients), eight types of hearing function could be identified using latent class analysis based on the audiogram of tinnitus patients (Langguth et al.). However, the authors recognized the limitations of the audiogram as a measure of hearing sensitivity, a factor contributing to increased interest in other measures, such as speech discrimination. In this regard, three studies found that tinnitus interferes with speech comprehension, consistent with dysfunctional central auditory processing in subjects with tinnitus (Gilles et al.; Ivansic et al.; Vielsmeier et al.). Reaction to external sounds, such as misophonia—the dislike of sound, also contributes to the heterogeneity of the tinnitus population (Palumbo et al.). A role for learning in annoyance and strong negative reactions to sound was emphasized as well as the similarity to synesthesia. Overall, these studies illustrate the wide variation in relationships between tinnitus and hearing loss as well as sensitivity to sound, contributing to the overall heterogeneity.

From a psychological perspective, it is of utmost importance to identify psychological features contributing to tinnitus and its heterogeneity. Several studies rose awareness for the core role of psychological features that have to be considered in tinnitus patients (e.g., stress, depression, alexithymia, cognition) in tinnitus (Alsalman et al.; Brüggemann et al.; Trevis et al.;  Wielopolski et al.). Wallhausser-Franke et al. repeatedly collected information on tinnitus perception among incident tinnitus patients and found that only one in 10 patients had complete remission after 6 months, while voiced complaints were stable in the majority of patients, and tinnitus-related distress worsened in 30% of tinnitus patients with depression at onset. Analyzing a case series data from Germany, tinnitus patients with comorbid headache (*n* = 193) were found to have had a lower quality of life compared to those without comorbid headache (*n* = 765), and greater painful sensation to loud sounds, vertigo, pain, and depressive symptoms (Langguth et al.). Interestingly, cluster analyses using data from 1,783 patients revealed two cluster solutions with clearly different characteristics, however with poor stability, suggesting that the tested tinnitus population comprised a continuum rather than a number of clearly defined subgroups (van den Berge et al.).

## Chapter 4: Use of Neuroimaging, Questionnaires and Mobile Instruments in Understanding Tinnitus Heterogeneity

Neuroimaging studies of individuals with tinnitus published in this research topic further suggest that tinnitus is accompanied by both structural (Allan et al.) and functional (Chen et al.; Chen et al.) brain changes in a distributed network of auditory and non-auditory brain regions. Structurally, a decrease in cortical thickness for the tinnitus group in the left superior frontal gyrus and a decrease in cortical volume with hearing loss in left Heschl's gyrus was found, while no changes were observed in the subcallosal region (Allan et al.) Functionally, a meta-analysis including nine resting-state neuroimaging studies shows in tinnitus patients an increased brain activity in the insula, middle temporal gyrus, inferior frontal gyrus, parahippocampal gyrus, cerebellum posterior lobe, and right superior frontal gyrus, when compared to controls (Chen et al.). These structural and functional alterations go together with an aberrant brain network architecture featuring a disrupted connectivity in non-auditory regions, especially the prefrontal cortex (Chen et al.), which findings were further confirmed by sound-evoked fMRI (Davies et al.) and auditory steady state responses (Engel et al.). Brain alterations in the theta and gamma bands were found in the auditory regions of tinnitus patients by magnetoencephalography (Lau et al.) and electroencephalography (Song et al.). Interestingly, these effects were no longer observed when excluding subjects with mental health comorbidities, strongly supporting the psychological burden in such alterations (Lau et al.). On the other hand, after cochlear implantation in single-sided deafness, an overall decrease in cortical activity and functional connectivity was seen in patients with tinnitus, likely due to dynamic peripheral reafferentation rather than cortical plastic changes (Song et al.). Taken together, these findings imply that the neural mechanisms underlying different subtypes of tinnitus share similar features when compared to controls. Future studies comparing subtypes may identify signatures specific to these subtypes.

Evaluation of tinnitus pitch and loudness was tested using two different methods, namely Touchscreen and Stand-alone, which both rapidly provided reliable measures (Hébert and Fournier). In the absence of any effective objective measures in humans, questionnaires have been supporting the assessment of tinnitus and its associated burden. Recent developments have prompted the translation and adaptation of several existing English language questionnaires into Swedish, Polish, and Italian (Müller et al.;  Moschen et al.; Wrzosek et al.). New questionnaires to evaluate auditory processing deficits have also been created (Diges et al.). Each of these questionnaires assesses a particular aspect of tinnitus (e.g., tinnitus-associated distress, anxiety, stress, depression, and life quality among others). Moreover, in order to facilitate a more holistic clinical evaluation, a visualization tool consisting of radar plots has been proposed by Schlee et al.

This special issue accommodates four papers on the potential of mobile crowdsensing for the support and empowerment of tinnitus patients, three of which promote the use of active patient participation and report on findings acquired with Ecological Momentary Assessments (EMA) (Probst et al.; Probst et al.; Schlee et al.). The extent to which smart devices, as well as more conventional internet-based solutions, have been taken up clinically for tinnitus diagnostics, treatment and self-help solutions has been reviewed by Kalle et al. The amounts of data collected interactively and unobtrusively through the smart devices call for data mining solutions for knowledge extraction toward the medical researchers and for adaptive models that adjust to the within-day and across time variability of patients' symptoms and needs.

## Chapter 5: Therapy

The heterogeneity of tinnitus may explain why treatment outcomes are so variable. In most clinical trials in which a given therapeutic intervention is investigated, treatment results vary considerably across patients as well from trial to trial, even if the same treatment is investigated. This may be partly due to variability in the methodology for patient assessment and outcome measurement. In their paper, Londero and Hall stress the relevance of standards for the reporting of outcome measurements. This is also a precondition for the development of valid treatment guidelines since the current ones vary considerably (Fuller et al.)

Sound therapy is an umbrella term that includes any form of acoustic stimulation used to increase the engagement of auditory system to offer tinnitus relief. The existing research demonstrates that even when attempting to personalize sound therapy, the complex combination of factors tinnitus patients present with is overlooked, and instead focus is made on one aspect such as pitch, maskability, hearing loss, sound preference or psychosocial factors (Searchfield et al.) A randomized controlled trial using cross-over study design and mixed methods revealed the significance of broadband noise compared to nature sound administration for reducing tinnitus (Durai and Searchfield). Exploratory studies such as the one conducted by Neff et al. found amplitude modulated sounds presented near tinnitus pitch to be more effective in tinnitus suppression, something that was more evident immediately after the stimulus offset. The past two decades have witnessed the commercialization of novel sound-based technologies, one such example is Acoustic CR Neuromodulation. This is a safe, well tolerated research tool which results in reduction of tinnitus symptoms for some patients, likely due to desynchronizing effects (Wegger et al.) However, the evidence for this technique to become a clinical tool is yet insufficient. Haller and Hall recommended that controlled trials on Acoustic CR Neuromodulation should include a well-characterized placebo group. Two studies investigated the neurophysiological mechanism underlying the effectiveness of sound therapy. Krick et al. used fMRI to show that the Heidelberg Neuro-Music Therapy (HNMT) decreases tinnitus-related distress by increasing the brain's default-mode network (DMN) activity. HNMT shifts the attention of tinnitus patients from the auditory phantom percept toward visual cues, a process involving an increased activity in the angular gyrus (Krick et al.). The advancement in sound based technological options, participation of industry and community partners along with emphasis of exploring the mechanism of effect is taking this management tool toward exciting horizon.

Cochlear implantation is one of the most successful therapeutic approaches for the control of tinnitus. However, it is only applicable for patients with uni- or bilateral deafness. Two articles of this special issue confirm the positive influence of cochlear implantation on the subjectively perceived tinnitus loudness and the tinnitus associated psychological burden (Bruggemann et al.; Knopke et al.). The positive aspects of cochlear implantation on tinnitus suppression might be interpreted as an effect of electrical stimulation of the auditory nerve, something that was tested via the electrical stimulation of the cochlea through the external auditory canal. Another study on cochlear implantation performed a sensitive analysis on the tinnitus improvement after surgery: Servais et al. found differential improvements for the perceptive and the reactive aspects of tinnitus. Tinnitus loudness was significantly reduced following CI surgery, while reduction of tinnitus-related distress, depression and anxiety did not reach statistical significance (Servais et al.). Two articles by Mielczarek et al. show that non-invasive electrical stimulation of the outer ear canal is able to induce neuroplastic changes in the auditory system suggestive of tinnitus improvements (Mielczarek et al.; Mielczarek et al.).

Brain stimulation is also an area of active research as a potential therapeutic tool. Three studies aimed at improving the efficacy of repetitive transcranial magnetic stimulation (rTMS). Whereas peripheral stimulation as an add-on to cortical stimulation did not show beneficial effects (Vielsmeier et al.), the individualization of stimulation parameters has revealed relatively promising results (Kreuzer et al.). In a case study, the effects of combined treatment with rTMS and cognitive behavioral therapy (CBT) on tinnitus and insomnia symptoms were reported (Richter et al.). CBT was in fact shown to be effective in a multidisciplinary day clinic (Ivansic et al.). Transcranial direct current stimulation (tDCS) is another non-invasive method for inducing neuroplastic changes by applying electrical currency to the brain. The article of Rabau et al. presents different possibilities of cathode placement, however, without being able to evidence a superiority of a certain position over another.

There is still no pharmacological treatment for tinnitus available. A pilot study presented the benefits of the hormone oxytocin on tinnitus loudness (Azevedo et al.). Another study identified a potential modulator of Acetylcholine receptors (Ach), that could have an impact on tinnitus treatments that are mediated via Ach transmission such as vagus nerve therapy (Bojić et al.). Figueiredo et al. found a higher prevalence of arterial hypertension in subjects with tinnitus in comparison to non-tinnitus controls, and proposed that treatment against hypertension with diuretics, ACE inhibitors or calcium channels blockers, could have had a role on tinnitus pathophysiology.

## Summary: Tinnitus Is a Complex Chronic Disorder

Complex disorders are caused by a combination of genetic and environmental factors. Within this research topic we have accumulated evidence for the genetic and the environmental influence on the development of tinnitus. Studies on genetic factors in tinnitus have been published in this research topic and point toward a significant genetic component, at least in some subtypes of tinnitus. As an example, the heritability in men with bilateral tinnitus was estimated with 0.68, while the heritability of unilateral tinnitus is estimated much lower with 0.27 (Cederroth et al.; Maas et al., 2017). The most obvious environmental influence for the development of tinnitus is the exposure to loud sounds that can lead to damage of the hearing system and finally trigger tinnitus (Eggermont). Besides noise trauma, other examples for environmental influences are the exposure to neurotoxic drugs such as cisplatin (Eggermont) or injuries that can lead to Temporo-Mandibular Disorders, which can also cause tinnitus (Haider et al.). Furthermore, genetic contributions for the treatment response of the patient have been hypothesized in this research topic and possible scientific approaches are discussed (Lopez-Escamez et al.). The complex interaction between genetic and environmental factors for the generation of tinnitus are currently unknown. Tinnitus research has just begun to address this complex issue, which may then help in the classification of subtypes.

Because there is a lack of effective treatments, the tinnitus condition remains chronic in the majority of cases. Even though spontaneous remissions exist, the percentage of cases is low. A study by Wallhäusser-Franke et al. in this research topic focused on acute tinnitus patients and found that only 11% of them reported a complete remission of tinnitus after 6 months. The remaining 89% of patients were classified as chronic (Wallhausser-Franke et al.). But this chronic condition does not necessarily mean that patients are perceiving the tinnitus permanently at the same intensity at all times. After 6 months, the majority of patients report an intermittent tinnitus. This has been supported by other studies from this Topic using a smartphone app to investigate the moment-to-moment variability of tinnitus (Schlee et al.) and to assess the circadian variability of tinnitus loudness (Probst et al.). The underlying mechanisms for this variability in the tinnitus percept is currently being investigated, but it is likely that exogeneous as well as endogenous factors influence the perception of tinnitus. Using magnetoencephalography, even the tinnitus-associated brain activity in the resting state was found to be variable over time (Schlee et al., 2014). These temporal variations of tinnitus add a fourth dimension to the already complex disorder of tinnitus. A better understanding of the chronobiological mechanisms involved in tinnitus might help the development of effective treatments, as recent studies reveal the importance of the chronotype in response to specific treatments of neurological disorders (McCarthy et al., 2018).

The medical condition of tinnitus is a complex and chronic disorder that holds a lot of open and challenging question for future research. In order to unravel this complexity, the combined effort of multiple scientific disciplines is needed—as evidenced in this research topic. The intellectual challenge for future tinnitus research will be to master this inter-disciplinary approach, to bring insights, expertise, and techniques from a range of academic and clinical specialties. Therefore, new ways of formal education at the universities will be needed in order to build up a new generation of researchers equipped with the skills to perform such inter-disciplinary research and further dig in the heterogeneity of this complex disorder. New concepts for such education are already at the horizon and the European School for Interdisciplinary Tinnitus research (ESIT), one of the first EU-funded tinnitus research, published their training curriculum in this research topic (Schlee et al.).

## Author Contributions

All authors listed have made a substantial, direct and intellectual contribution to the work, and approved it for publication.

### Conflict of Interest Statement

The authors declare that the research was conducted in the absence of any commercial or financial relationships that could be construed as a potential conflict of interest.
